# Visualization of HIV-1 RNA Transcription from Integrated HIV-1 DNA in Reactivated Latently Infected Cells

**DOI:** 10.3390/v10100534

**Published:** 2018-09-30

**Authors:** Obiaara B. Ukah, Maritza Puray-Chavez, Philip R. Tedbury, Alon Herschhorn, Joseph G. Sodroski, Stefan G. Sarafianos

**Affiliations:** 1 CS Bond Life Sciences Center, University of Missouri, Columbia, MO 65201, USA; obif29@mail.missouri.edu (O.B.U.); mnpkx3@mail.missouri.edu (M.P.-C.); 2 Department of Molecular Microbiology & Immunology, University of Missouri School of Medicine, Columbia, MO 65212, USA; philip.tedbury@emory.edu; 3 Division of Infectious Diseases and International Medicine, Department of Medicine, University of Minnesota, Minneapolis, MN 55455, USA; aherschh@umn.edu; 4 Department of Cancer Immunology and Virology, Dana-Farber Cancer Institute, Department of Microbiology and Immunobiology, Harvard Medical School, Boston, MA 02115, USA; joseph_sodroski@dfci.harvard.edu; 5 Department of Immunology and Infectious Diseases, Harvard School of Public Health, Boston, MA 02115, USA; 6 Department of Biochemistry, University of Missouri, Columbia, MO 65211, USA

**Keywords:** human immunodeficiency virus, in situ hybridization, latency, reactivation

## Abstract

We have recently developed the first microscopy-based strategy that enables simultaneous multiplex detection of viral RNA (vRNA), viral DNA (vDNA), and viral protein. Here, we used this approach to study the kinetics of latency reactivation in cells infected with the human immunodeficiency virus (HIV). We showed the transcription of nascent vRNA from individual latently integrated and reactivated vDNA sites appearing earlier than viral protein. We further demonstrated that this method can be used to quantitatively assess the efficacy of a variety of latency reactivating agents. Finally, this microscopy-based strategy was augmented with a flow-cytometry-based approach, enabling the detection of transcriptional reactivation of large numbers of latently infected cells. Hence, these approaches are shown to be suitable for qualitative and quantitative studies of HIV-1 latency and reactivation.

## 1. Introduction

The human immunodeficiency virus type 1 (HIV-1) latent reservoir is a major barrier to clearing the virus, and cure strategies attempting to reactivate and kill infected cells (the so called kick-and-kill methods) have struggled due to difficulties in efficiently reactivating transcriptionally silent proviruses [1,2,3,4]. The methods that are currently available for detection of the frequency of latently infected cells in HIV patients include (a) viral outgrowth assays, which underestimate the size of the latent reservoir due to failure to reactivate all latent cells [1,5,6,7,8,9,10], and (b) PCR amplification of HIV-1 viral DNA (vDNA), which can overestimate the size of the reservoir due to the amplification of replication-incompetent viruses [1,11,12,13,14,15,16,17,18,19,20,21]. Consequently, there is a need for sensitive methods that enable direct visualization and/or detection of HIV nucleic acid in latently infected cells. Such methods could allow us to better estimate the latent reservoir size, and to identify and characterize potential latency-reversing agents (LRAs).

Fluorescent branched DNA in situ hybridization (bDNA ISH) has been used for the detection of host and viral RNA (vRNA) in cell lines and peripheral blood mononuclear cells (PBMCs) [22]. It has also been used for the detection of simian immunodeficiency virus (SIV) vDNA and vRNA in tissue cross-sections from chronically infected rhesus macaques [23], and to follow the reactivation of HIV-1 from latency [24]. The combination of protein and RNA staining has been shown to enhance sensitivity and specificity when screening large numbers of cells for rare reactivated cells [24,25,26,27].

Here we augmented our recently developed highly sensitive assay [28], which combines fluorescence-based bDNA ISH technology with immunofluorescence detection for the simultaneous assessment of vRNA, vDNA, and Gag proteins with flow cytometry. Using a combination of microscopy and flow cytometry, before and after transcriptional reactivation, we were able to follow the dynamics and kinetics of transcriptional reactivation of HIV-1 in individual latently infected cells. We found that vRNA imaging is suitable for the quantitative analysis of the extent and kinetics of reactivation, and allowed a more rapid determination of reactivated cells than protein imaging. Imaging of vRNA with protein also permits the identification of cells that are competent for transcription but not translation; such cells are a component of the reservoir of defective proviruses, which, despite being defective for infectious virus production, may contribute to HIV-1 pathogenesis.

## 2. Materials and Methods

### 2.1. Cell Culture and Virus

JLat 10.6 cells (NIH AIDS Reagent Program catalog #9849) are Jurkat-derived human T cells that are latently infected with the packaged retroviral construct HIV-R7/E-/GFP, which is full length HIV-1 minus *env*, minus *nef* [29]. J1.1 cells (NIH AIDS Reagent Program catalog #1340) are also Jurkat-derived human T cells, but are latently infected with LAV, a full-length HIV-1 isolate [30]. Both cell types were cultured in RPMI 1640 medium (Gibco, Waltham, MA, USA), supplemented with 10% heat-inactivated fetal bovine serum (FBS) and 2 mM l-glutamine (Gibco), in a humidified incubator at 37 °C with 5% CO_2_.

For primary cell experiments, CD4^+^ T cells were isolated from PBMCs using EasySep™ Human CD4^+^ T Cell Enrichment Kit (STEMCELL Technologies, Vancouver, BC, Canada), following the manufacturer’s instructions. T cells were cultured and stimulated in RPMI 1640 medium supplemented with 10% heat-inactivated FBS, 2 mM l-glutamine, 60 U/mL IL-2, and 25 µL/mL ImmunoCult Human CD3/CD28/CD2 T-Cell Activator (STEMCELL Technologies), for 3 days prior to infection. Cells were maintained in a humidified incubator at 37 °C with 5% CO_2_.

Single cycle infections were performed with viruses that were prepared using HIfate-E, a dual fluorescence reporter that will be fully characterized elsewhere (Herschhorn & Sodroski). This system is based on a previously described construct [31] with several improvements, and expresses green fluorescent protein (GFP) from a constitutively active elongation factor 1a-human T cell leukemia virus type I (EF1a-HTLV-I) composite promoter, and E2-Crimson (a far-red fluorescent protein) from the HIV-1 5′LTR promoter. Cells were infected as previously described [29]. Briefly, activated CD4^+^ T cells with virus were spinoculated into 6-well plates with RPMI, IL-2, ImmunoCult Human Activator, and 8 µg/mL polybrene for 1 h at 1200× *g* at 20 °C. After spinoculation, cells were placed at 37 °C and the medium was replaced after 4 h with RPMI, IL-2, and ImmunoCult Human Activator. CD4^+^ T cells were sorted 4 days post infection with a Beckman Coulter MoFlo XDP (Indianapolis, IN, USA).

### 2.2. Antibodies and Compounds

A mouse monoclonal anti-p24 antibody raised against the p24/capsid domain of Gag [32,33] and a goat anti-mouse secondary antibody conjugated to Alexa Fluor 488 (Invitrogen) were used to detect Gag after cell fixation. Compounds used for latency reversal in latent cell lines included: (1) 10 ng/µL TNF-α (Genscript, Piscataway, NJ, USA), (2) 81 nM phorbol 12-myristate 13-acetate with 1.34 µM ionomycin (PMA/I) (eBioscience, San Diego, CA, USA), (3) 100 nM romidepsin (FK228; Cayman Chemical, Ann Arbor, MI, USA), (4) 1 µM JQ1 (Cayman Chemical), and (5) 10 µM 3-deazaneplanocin A (DZNep; Cayman Chemical). Cells were treated for 4–24 h, depending on the assay performed.

### 2.3. Nucleic Acid Probes

All probes were purchased from Advanced Cell Diagnostics (ACD, Newark, CA, USA). To detect HIV-1 RNA two anti-sense probes were used (Supplementary Table S1); the first probe set (PS-1) targets non-*gag*-*pol* regions coding envelope and accessory proteins (HIV RNA anti-sense probe-1, (probe channel C2)), and the second probe set (PS-2) targets the *gag* coding region of HIV-1 genomic RNA (HIV RNA anti-sense probe-2, (probe channel C1)) [22]. For HIV-1 DNA detection, a third probe set (PS-3) targeting the *gag*-*pol* coding region was used to avoid binding to viral mRNA (HIV DNA sense probe-3, (probe channel C1)).

### 2.4. In Situ vRNA Detection via Microscopy

HIV-1 RNA in cells was probed using RNAscope reagents (Advanced Cell Diagnostics). The manufacturer’s protocol was followed with some modifications as described by Puray-Chavez et al. [28,34]. Specific pre-designed antisense probes that recognize the HIV-1 RNA were used. Following staining, coverslips were mounted on slides using Prolong Gold Antifade. Probes are described in Supplementary Table S1.

### 2.5. Simultaneous In Situ vRNA and vDNA Detection via Microscopy

For co-staining of vRNA and vDNA samples, the hybridization of target probes occurred simultaneously, and the protocol was then followed as described for vRNA staining.

### 2.6. Immunostaining for Microscopy

Protein staining was performed after in situ hybridization (ISH). Coverslips were blocked with 1% bovine serum albumen (BSA) and 10% FBS in PBS containing 0.1% Tween-20 (PBST) at room temperature for 1 h. Gag was then probed with a monoclonal antibody raised against the p24/capsid domain [32,33] that was diluted 1:2000 in PBST supplemented with 1% BSA, and incubated at room temperature for 1 h. Samples were washed twice in PBST at room temperature for 10 min with rocking. Fluorescently labelled secondary antibody was used at 1:2000 and incubated at room temperature for 1 h, and then the samples were washed once with in PBST at room temperature for 10 min with rocking. Nuclei were stained using DAPI and the coverslips were mounted as described previously.

### 2.7. Imaging and Imaging Quantification

Unless otherwise stated, images were taken with a Leica TCS SP8 inverted confocal microscope equipped with a 63×/1.4 oil-immersion objective, and a tunable supercontinuum white light laser. The excitation/emission bandpass wavelengths used to detect DAPI, Alexa 488, ATTO 550, Alexa 568, and Alexa 647 were set to 405/420–480, 488/505–550, 550/560–610, 568/580–630, and 647/655–705 nm, respectively. For time course experiments, fields of view were selected randomly based on the DAPI signal. Images were taken of the DAPI, RNA, and DNA staining (10 images per sample using the 63× objective). The percentage of cells positive for vRNA (PS-2) were quantified using Gen5 software (Biotek, Winooski, VT, USA), and the number of vRNA^+^ cells for each image set was divided by the total number of cells in the image to determine the average number of vRNA^+^ cells per LRA treatment.

Z-stack movies were generated using the Leica Application Suite and edited using Fiji [35].

### 2.8. In Situ vRNA Detection via Flow Cytometry Analysis

HIV-1 RNA in cells was probed using PrimeFlow reagents (Affymetrix, Santa Clara, CA, USA). The protocol was performed over 2 days following the manufacturer’s recommendations. Following staining, the samples were resuspended in the desired amount of fresh Storage Buffer, then analyzed on a BD Accuri C6 flow cytometer (San Jose, CA, USA).

### 2.9. Statisitcal Analyses

Analyses were performed and graphs plotted using Graphpad Prism 6 (La Jolla, CA, USA). For multiple comparisons, a one-way ANOVA was performed with Tukey’s post-test for significant differences. Where multiple comparisons were made to a single control sample, Dunnett’s post-test was used.

## 3. Results

### 3.1. Detection of Protein and Nucleic Acids in a Model Latent Cell Line

**Figure 1:**
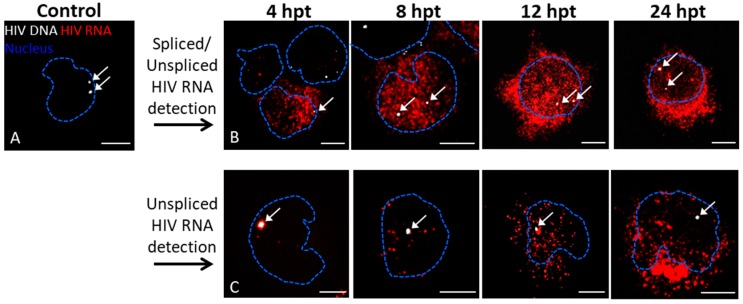
Branched DNA (bDNA) in situ hybridization reveals time-dependent differences between spliced and unspliced vRNA transcription and export from the nucleus. Untreated JLat 10.6 cells (**A**) and JLat 10.6 cells treated with tumor necrosis factor-α (TNF-α; **B** and **C**). Cells were processed for detection of total (spliced and unspliced) vRNA (**B**) or unspliced vRNA (**C**). Z-stacks were captured using a Leica SP8 confocal microscope with a 63×/1.4 oil-immersion objective. Each panel displays the maximum projection or the combination of each Z-stack layer for visualization of total vRNA produced per cell. Nuclei were stained with DAPI; the nuclear outline is indicated with a dotted blue line. White arrows indicate HIV-1 proviruses. Scale bars are 5 µm.

For initial trials of nucleic acid staining to assess reactivation from latency, we used a well-characterized latently infected cell model, JLat 10.6 [29]. Under standard culture conditions, these cells do not produce HIV-1; however, following treatment with LRAs, activation of HIV-1 transcription can be followed by visualization of GFP production [29]. Thus, JLat 10.6 cells provide a convenient and efficient measure of transcriptional reactivation (Supplemental Figure S1). However, this approach and other protein detection methods depend on translation and folding of the protein. In our experiments, GFP fluorescence was observed at 8 h post reactivation, but not at 4 h. To permit the monitoring of reactivation at earlier time points, we sought to visualize HIV-1 vRNA transcription. JLat 10.6 cells were reactivated using TNF-α, a cytokine that is known to induce latent HIV-1 activation through NFkB signaling [36]. Subsequently, we followed the dynamics of viral transcription through the detection of nascent vRNA with a selection of probes (Supplementary Table S1), including probes targeting multiply and singly spliced vRNA (PS1), or a combination of unspliced and spliced vRNA (PS2). The probes and experimental conditions were designed to allow simultaneous detection of spliced and unspliced vRNA in individual cells. JLat 10.6 cells were treated with LRAs for 4, 8, 12, or 24 h, then fixed and stained by fluorescent bDNA ISH. Spliced vRNA was abundant and could be detected outside the nucleus as early as 4 h post treatment (hpt), before accumulation or nuclear export of unspliced vRNA (Figure 1 and Supplemental Movie S1). Over time, both spliced and unspliced RNA accumulated, first in the nucleus, and then following nuclear export, in the cytoplasm (Figure 1 and Supplemental Movie S2). The more pronounced early accumulation of spliced vRNA is consistent with prior reports of more rapid accumulation of spliced vRNA than unspliced vRNA in T cells [37]. Similarly, the accumulation of vRNA in the nucleus is driven by the requirement for Rev to export unspliced vRNA via the interaction with a structured RNA known as the Rev response element [38,39]. Rev itself is translated from a multiply spliced vRNA [40]. At 24 hpt with TNF-α, a very small number of vRNA-containing cells did not produce GFP (Supplemental Figure S2). Hence, this approach could be used to identify cells that become competent for transcription but not translation of authentic viral proteins, after reactivation with LRAs.

To visualize the reactivation of transcription from specifically labeled HIV-1 proviral DNA, we targeted the *gag-pol* complementary region of the negative strand of HIV-1 (PS3). We were able to determine that JLat 10.6 cells contain two copies of proviral HIV-1 in the nucleus (Figure 1A,B); in some cases, only one integration site is apparent, which is likely a consequence of the second site lying in an out-of-focus plane, or incomplete labeling of the integrated proviruses. Hence, we were able to demonstrate the reactivation of transcription from individual HIV-1 proviruses in latently infected cells. Ongoing efforts focus on the isolation of individual cells, characterization of the locations of proviral integration, and factors that influence proviral activity.

### 3.2. Quantifying the Efficacy of Latency Reactivating Agents

**Figure 2:**
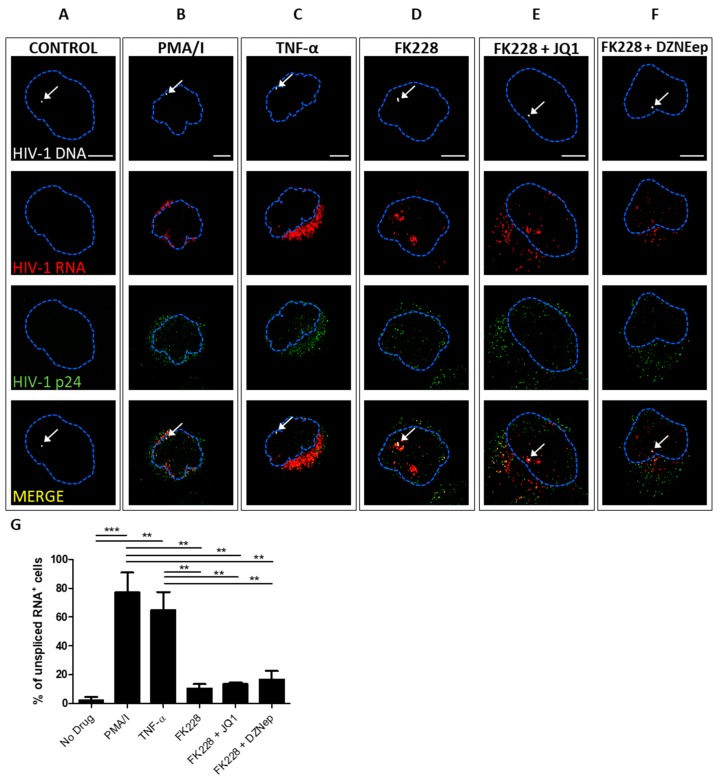
bDNA in situ hybridization together with immunocytochemistry allows assessment of vRNA and Gag after reactivation with LRAs. Untreated J1.1 cells (**A**) and J1.1 cells treated with TNF-α (**B**), PMA/I (**C**), romidepsin (**D**), romidepsin and JQ1 (**E**), or romidepsin and DZNep (**F**) for 8 h, then processed for detection of unspliced vRNA, vDNA, and Gag protein. Z-stacks were captured using a Leica SP8 confocal microscope with a 63×/1.4 oil-immersion objective. Each panel displays the Z-stack layer in which vDNA was visualized, to reveal associations between vDNA, vRNA, and Gag in the nucleus. Nuclei were stained with DAPI; the nuclear outline is indicated with a dotted blue line. White arrows indicate sites of vDNA integration. Scale bars are 5 µm. (**G**) The percentage of cells staining positive for unspliced RNA at 8 h post-treatment for each of the latency reactivating agents (LRAs) was averaged from three independent experiments and plotted ± the standard error of the mean. ** *p* ≤ 0.01, *** *p* ≤ 0.001 as determined by Tukey’s multiple comparison test.

We also used this method to establish a microscopy-based approach that allows the identification and validation of compounds that effectively reactivate latently infected cells. Experiments were carried out with J1.1 cells, which, unlike JLat 10.6 cells, produce infectious virus after reactivation with LRAs [30]. We tested the reactivation of J1.1 cells using the following LRAs or combinations of LRAs for 8 h: (1) TNF-α; (2) PMA/I [41,42], (3) FK228 (romidepsin, a histone deacetylase inhibitor) [43,44], (4) FK228 with JQ1 (a bromodomain and extra-terminal motif (BET) inhibitor) [45,46,47], and (5) FK228 with DZNep (a histone methyltransferase inhibitor) [48,49] (Figure 2). Following reactivation, cells were imaged for unspliced vRNA, vDNA, and Gag protein [32,33]. Transcription and export of unspliced vRNA can be seen 8 hpt with all LRAs and LRA combinations except FK228, which showed vRNA restricted primarily to the nucleus (Figure 2D), possibly suggesting less potent reactivation of HIV-1. The use of immunocytochemistry in combination with ISH showed that activated cells were typically positive for both vRNA and Gag, for all of the LRAs used (Figure 2). To address the efficiency of HIV-1 reactivation by the different LRAs, cells were reactivated as described above and stained for unspliced RNA, and randomly selected fields were analyzed to identify the percentage of activated cells (Figure 2G). Consistent with previous reports, TNF-α and PMA/I were found to be the most potent LRAs in our experiments.

**Figure 3:**
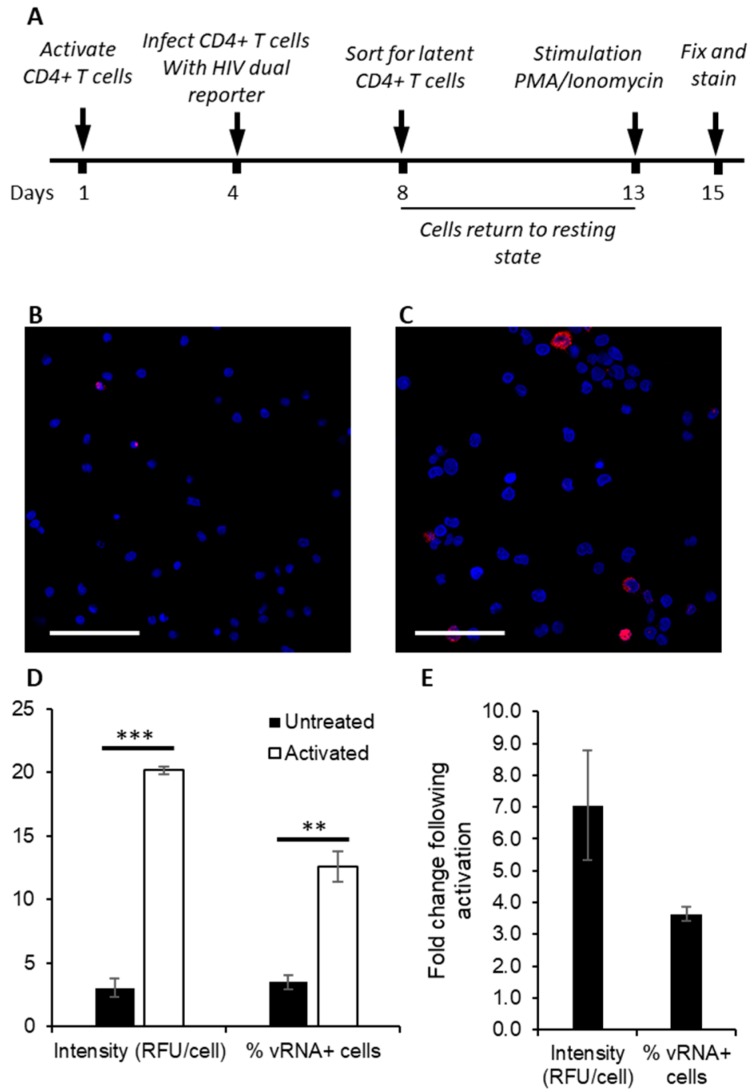
Fluorescent branched DNA in situ hybridization (bDNA ISH) can be used to assess reactivation from latency in primary CD4^+^ T cells. (**A**) CD4^+^ T cells were isolated from peripheral blood mononuclear cells (PBMCs) and activated for 3 days. Cells were infected with HIfate-E, a dual reporter virus, incubated for 4 days, and then the latent population was collected using fluorescence activated cell sorting (FACS). The latent cells were allowed to return to a resting state for 5 days and were subsequently activated with PMA/I for 2 days, then fixed and permeabilized prior to staining. ISH was performed for spliced and unspliced vRNA, and images of randomly selected fields were captured using a Leica SP8 confocal microscope with a 63×/1.4 objective. The number of cells analyzed per sample totaled 500–700. Representative fields of view show (**B**) unstimulated cells and (**C**) cells activated with PMA/I. Scale bars are 50 μm. (**D**) HIV-1 activation was measured as an increase in the fluorescent signal above background, expressed as relative fluorescence units (RFU) per cell, and as percentage of activated cells (positive for HIV-1 RNA). (**E**) Fold changes in fluorescence signal and percentage of activated cells following activation with PMA/I. Data from two independent experiments were plotted ± the standard error of the mean. ** *p* ≤ 0.01, *** *p* ≤ 0.001 as determined by Tukey’s multiple comparison test.

To further validate the utility of bDNA ISH for use in primary cells, we examined the reactivation of latent HIV-1 from infected primary cells. CD4^+^ T cells were isolated from PBMCs and infected with a dual color reporter virus, similar to constructs that have been reported previously [31,50]. The reporter virus expresses GFP under the control of a constitutively active EF1a-HTLV-I promoter, and E2-Crimson (far red) under the control of the HIV-1 5’LTR. Consequently, actively infected cells are double positive, while cells expressing GFP but not E2-Crimson are latently infected. Latently infected (GFP+/Crimson-) cells were collected by fluorescence-activated cell sorting (FACS), split into two samples, and one sample was treated with PMA/I to reactivate latent HIV-1. PMA/I is reportedly more effective than TNF-α for reactivation of latent HIV-1 in primary cells [51]. As there were not sufficient latent cells for FACS analysis, the cells were instead mounted on poly-l-lysine-treated coverslips and stained for spliced and unspliced vRNA using bDNA ISH. A larger number of vRNA positive cells were visible in reactivated cells than in those untreated (Figure 3B,C). Both total fluorescence signal and number of vRNA positve cells were quantified, revealing increases of ~7- and ~3.5-fold following activation (Figure 3D,E). Consistent with previous reports, these data suggest that bDNA ISH labeling of vRNA is an effective method for monitoring HIV-1 infection [23,52] and reactivation in primary cells [24,25].

### 3.3. Quantification of Latency Reactivation by Flow Cytometry

**Figure 4:**
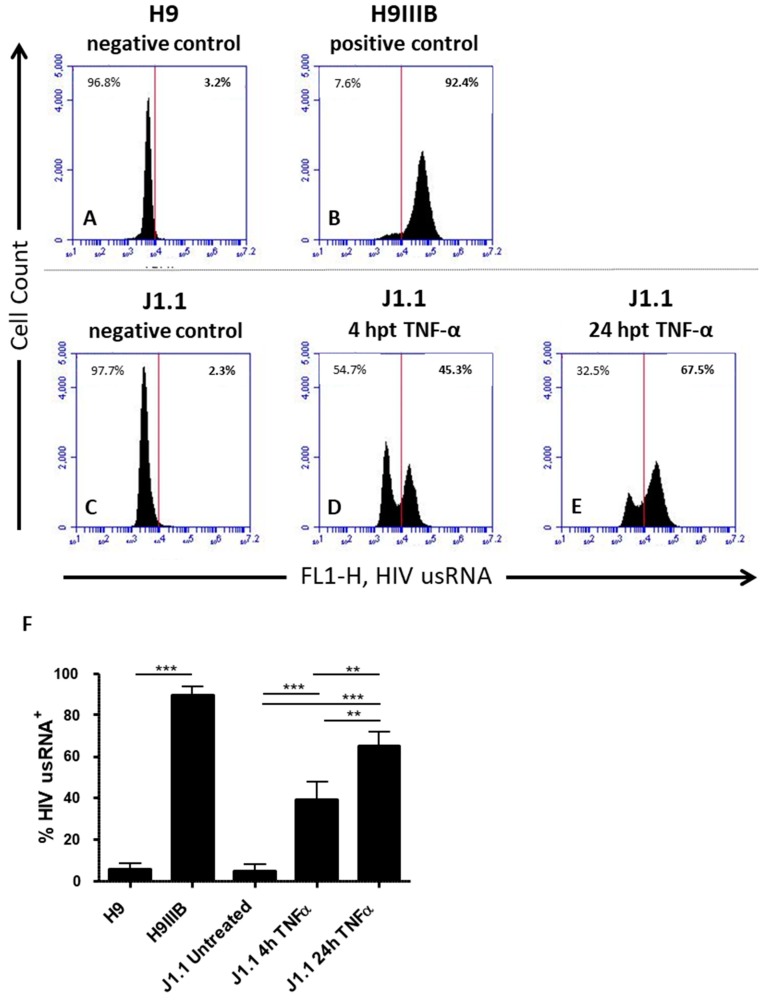
Untreated cells H9 (**A**), H9IIIB (**B**), and J1.1 cells (**C**), and J1.1 cells treated with TNF-α for 4 h (**D**) or 24 h (**E**) were fixed and permeabilized prior to staining. ISH was performed on samples and flow cytometry analysis with an Accuri C6 (Becton Dickinson, San Jose, CA, USA) was utilized to detect cells positive for HIV-1 unspliced vRNA fluorescently labeled with Alexa 488. The number of cells analyzed per sample totaled 3–3.5 × 10^4^ cells. (**F**) The percentage of activated cells was averaged from three independent experiments and plotted ± the standard error of the mean. ** *p* ≤ 0.01, *** *p* ≤ 0.001 as determined by Tukey’s multiple comparison test.

Lastly, we demonstrate that fluorescent bDNA ISH for detection of vRNA through microscopy can be enhanced as a screening tool for LRAs when used in combination with flow cytometry. While microscopy can provide mechanistic information about individual cells, flow cytometry can be used to screen thousands of cells for transcriptional reactivation in a matter of minutes [24] (Figure 4). For positive and negative controls, we probed for unspliced vRNA (PS-4) in uninfected H9 cells [53,54,55] (Figure 4A), and in their derivative cell line H9IIIB, which is chronically infected with the IIIB strain of HIV-1 [53,54,56] (Figure 4B). For flow cytometry, we used Primeflow (Affymetrix) bDNA ISH. As expected, we observed no HIV-1 vRNA signal with the uninfected H9 cells and a robust positive signal with the chronically infected H9IIIB cells (Figure 4A,B, respectively). Using J1.1 cells as our latency model, we screened untreated cells (Figure 4C) and cells treated with TNF-α at various time points (Figure 4D,E). These measurements revealed a time-dependent increase in the percentage of reactivated cells producing unspliced transcripts. At 4 hpt, ~45% of cells had been reactivated (Figure 4D,F), and the total percentage of reactivated cells increased to ~70% at 24 hpt (Figure 4E,F).

## 4. Discussion

We have established protocols for efficient multiplex detection of viral nucleic acids and viral proteins produced from individual integration sites, following reactivation in established models for latently infected cells. One surprise arising from the imaging of vDNA in addition to vRNA was the observation of two proviruses in JLat 10.6 cells. As these cells are reported to possess only one integrated provirus [57], we suspect that the second provirus is incomplete; alternatively, it may reside in a duplicated region of the genome or in a region that is not conducive to identification by deep sequencing approaches. Our LRA data demonstrate that staining vRNA is a viable alternative to staining protein, and can be measured at earlier time points. We also employed the method to verify the reactivation of latent virus in primary CD4^+^ T cells. In combination with earlier reports showing the utility of bDNA ISH to visualize infection by primary virus isolates, we feel this method has practical applications in the study of reactivation from latency in clinical samples [23,24,25,28]. Furthermore, as a practical consideration, the probes recognizing spliced and unspliced RNA produced a more robust signal, particularly at early time points, than those against unspliced RNA alone; this would likely provide superior sensitivity to the assay.

Overall, our data are consistent with findings of other groups, and show that these approaches may help measure the size of the latent reservoir more accurately, aid in determining the types of LRAs that most effectively reactivate latent virus, and help characterize the transcriptional and translational competence of reactivated cells [22,23,24,25,26]. It may also be possible to identify specific populations of cells harboring defective proviruses, such as those competent for transcription but not translation of authentic viral proteins. It is expected that variability in transcription and translation competence will be far greater in patient derived samples than in the clonal cell lines used in this study, as clinically-derived proviruses have been observed to vary widely in their completeness, many producing incomplete or aberrant RNAs [58,59]. It would also be desirable to be able to visualize proviral vDNA in latently infected clinical samples; however, at this time, the scarcity of these cells combined with the small foci of fluorescence make such approaches untenable. To permit analysis of these cells by flow cytometry or similar methods, further technological advances that improve the signal from a single molecule will be necessary. Nevertheless, the current technology may contribute towards a better understanding of HIV-1 latency.

## Supplementary Materials

The following are available online at http://www.mdpi.com/1999-4915/10/10/534/s1, Figure S1: JLat 10.6 cells produce GFP in a time-dependent manner, Figure S2: bDNA ISH and confocal microscopy revealed rare translation-incompetent cells, Table S1: Summary of Target Probe Sets, Video S1: Transcription of HIV-1 RNA in JLat 10.6 cells 4 h post reactivation, Video S2: Transcription and export of HIV-1 RNA in JLat 10.6 cells 12 h post reactivation.
