# Supplementary material for: Visualization of HIV-1 RNA Transcription from Integrated HIV-1 DNA in Reactivated Latently Infected Cells
Source: Viruses. 2018 Sep 30;10(10):534. doi: 10.3390/v10100534 (PMC6212899; doi:10.3390/v10100534)
Supplement: Supplementary file 1 [file viruses-10-00534-s001.zip › Supplemental Material_revised.docx]

**Supplemental Figures**


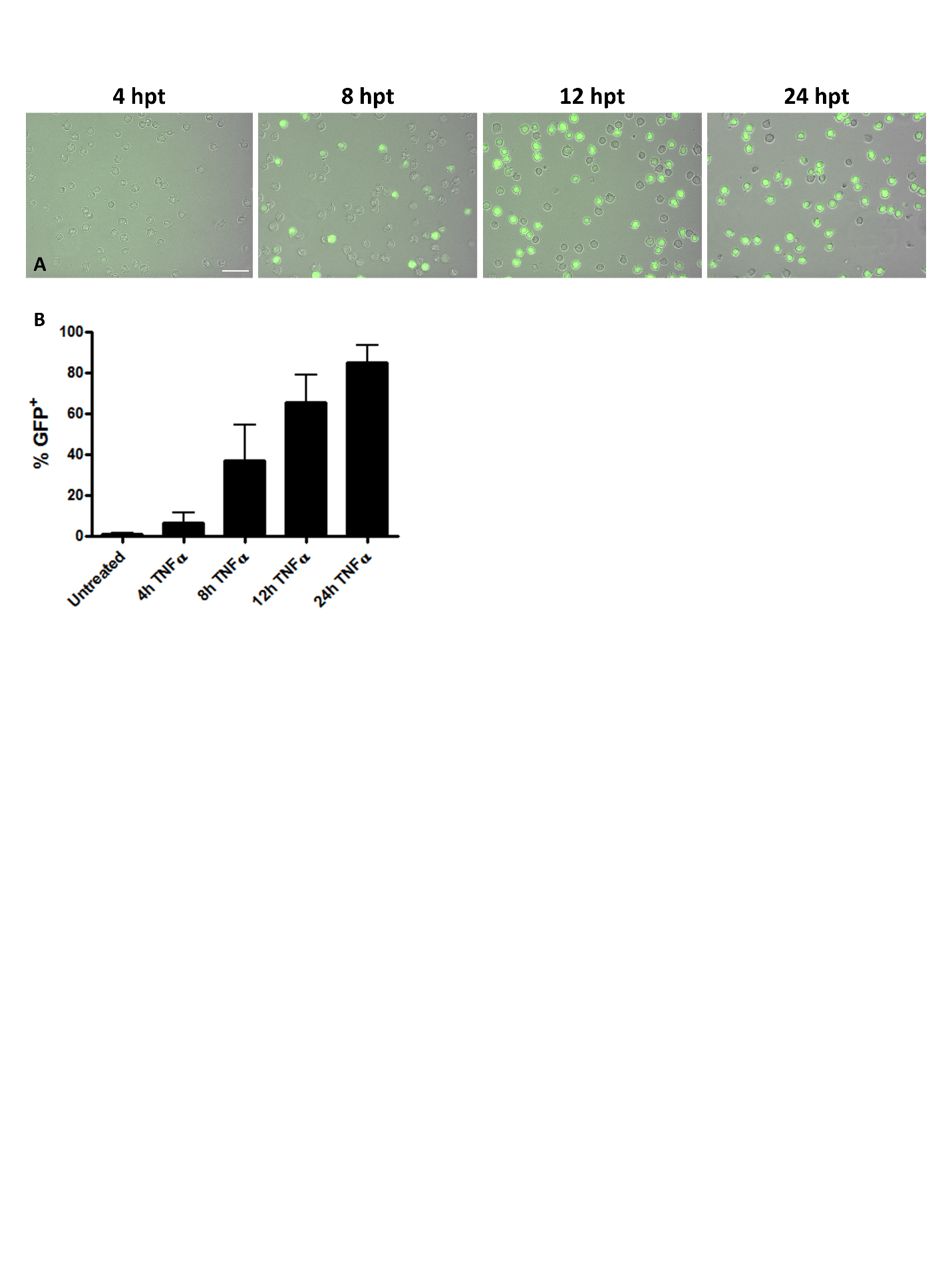


**B**

**Untreated**

**α**

**4h TNF**

**α**

**8h TNF**

**α**

**12h TNF**

**α**

**24h TNF**

**0**

**20**

**40**

**60**

**80**

**100**

***

***

**

**% GFP**

**+**

**Figure S1. JLat 10.6 cells produce GFP in a time-dependent manner.** (A) JLat 10.6 cells were treated with 10 ng/ml TNF-α for 4 h, 8 h, 12 h, or 24 h, and GFP production visualized with an Olympus IX81 microscope, using a 20x/0.45 objective. Images show a merge of the GFP channel a with phase contrast, to reveal GFP^-^ cells. Scale bar equals 50 µm. (B) The percentage of activated cells (GFP^+^) was averaged from 3 independent experiments and plotted +/- the standard error of the mean. **p ≤ 0.01, ***p ≤ 0.001 as determined by Dunnett’s Multiple Comparison Test.


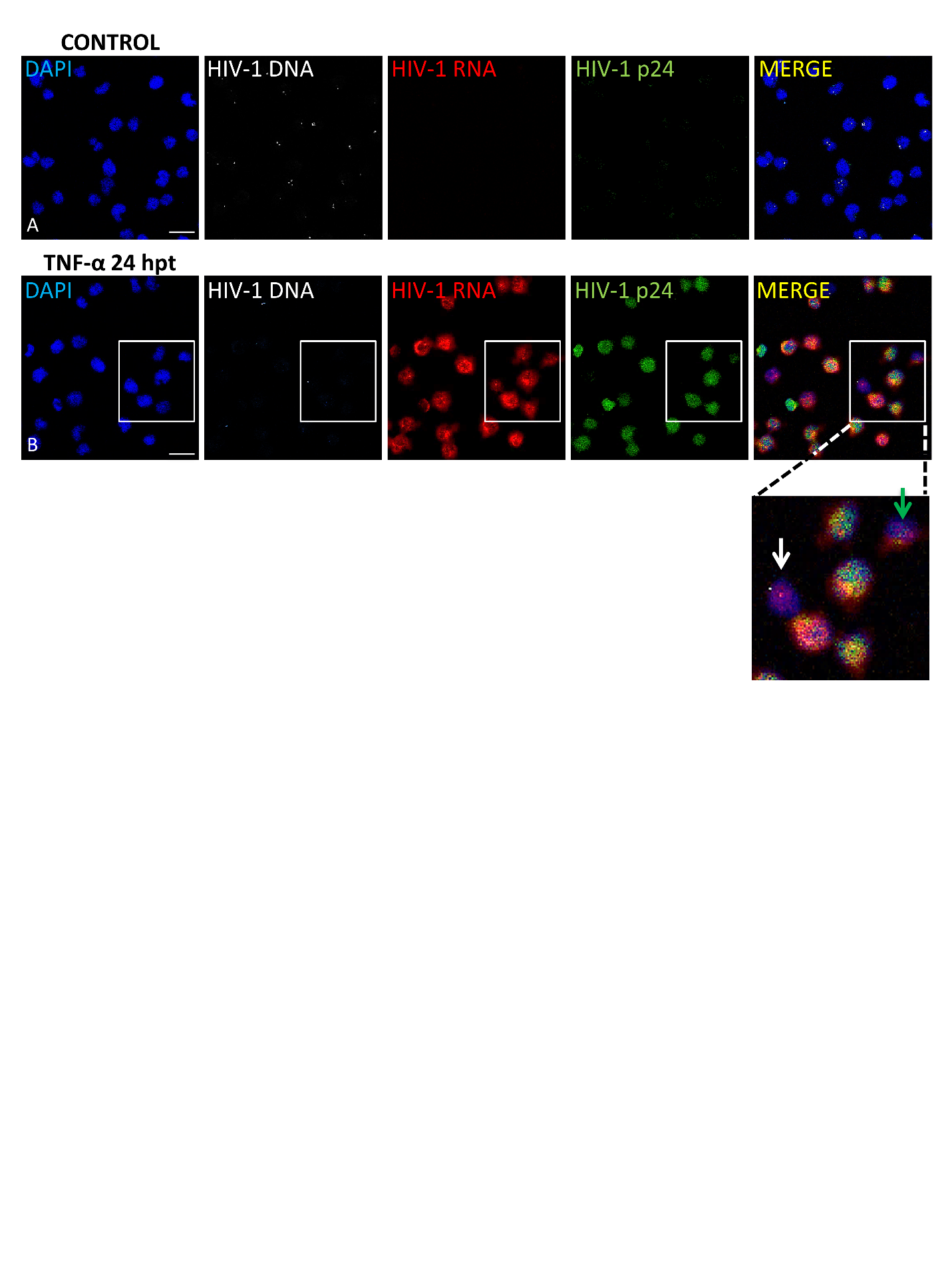


**Figure S2.** **bDNA ISH and confocal microscopy revealed rare translation-incompetent cells.** Untreated JLat 10.6 cells (A) or cells treated with TNF-α for 24 h (B), were fixed and stained as in Fig. 1.Cells were imaged with a Leica SP8 confocal microscope using a 20x/0.7 objective. A white arrow indicates a cell with no Gag expression, despite vRNA levels comparable to the cell indicated by a green arrow. In future work, it may be possible to use fluorescence-activated cell sorting to enrich these cells for further analyses. Scale bar equals 20 µm.

**Supplemental Movie 1. Transcription of HIV-1 RNA in JLat 10.6 cells 4 h post reactivation.** Z-stack of a JLat 10.6 cell following activation with TNF-α, allowing visualization of unspliced vRNA transcription from an integrated provirus (Figure 1, unspliced vRNA at 4 h). Moving through the cell in the Z-axis, the provirus is visible inside the nucleus, and, at this early time point, vRNA is also only visible inside the nucleus. A small amount of nascent vRNA can be seen in close proximity to the proviral DNA. Movie generated using Leica Application Suite and edited using Fiji.

**Supplemental Movie 2. Transcription and export of HIV-1 RNA in JLat 10.6 cells 12 h post reactivation.** Z-stack of a JLat 10.6 cell following activation with TNF-α. At this later time, unspliced vRNA in the cytoplasm is now visible in the cytoplasm, and distributed though the nucleus (Figure 1, unspliced vRNA at 12 h). Additionally, presumed nascent unspliced vRNA can be seen in close proximity to the integrated provirus. Movie generated using Leica Application Suite and edited using Fiji.

**Table S1.** **Summary of Target Probe Sets**

| **Probe Name** | **Probe Target** | **Reference Sequence**  **PubMed Accession #** | **Target Sequence** |
| --- | --- | --- | --- |
| PS1 | Multiply & singly spliced  HIV-1 RNA | [NC_001802.1](http://www.ncbi.nlm.nih.gov/nuccore/NC_001802.1) | Non *gag-pol* regions  (4988 - 9181) |
| PS2 | Unspliced HIV-1 RNA | [NC_001802.1](http://www.ncbi.nlm.nih.gov/nuccore/NC_001802.1) | *gag-pol* region  (801 - 1393) |
| PS3 | HIV-1 DNA | [NC_001802.1](http://www.ncbi.nlm.nih.gov/nuccore/NC_001802.1) | *gag-pol* region  (507 - 4601) |
| PS4 | Unspliced HIV-1 RNA | K03455.1 | *gag-pol* region  (1653 - 2658) |
